# Antiviral Cytokine Response in Neuroinvasive and Non-Neuroinvasive West Nile Virus Infection

**DOI:** 10.3390/v13020342

**Published:** 2021-02-22

**Authors:** Snjezana Zidovec-Lepej, Tatjana Vilibic-Cavlek, Ljubo Barbic, Maja Ilic, Vladimir Savic, Irena Tabain, Thomas Ferenc, Ivana Grgic, Lana Gorenec, Maja Bogdanic, Vladimir Stevanovic, Dario Sabadi, Ljiljana Peric, Tanja Potocnik-Hunjadi, Elizabeta Dvorski, Tamara Butigan, Krunoslav Capak, Eddy Listes, Giovanni Savini

**Affiliations:** 1 Department of Immunological and Molecular Diagnostics, University Hospital for Infectious Diseases “Dr Fran Mihaljevic”, 10000 Zagreb, Croatia; szidovec@gmail.com (S.Z.-L.); ivanahobby@gmail.com (I.G.); lgorenec@gmail.com (L.G.); 2 Department of Virology, Croatian Institute of Public Health,10000 Zagreb, Croatia; irena.tabain@hzjz.hr (I.T.); maja.bogdanic11@gmail.com (M.B.); 3 Department of Microbiology, School of Medicine, University of Zagreb, 10000 Zagreb, Croatia; 4 Department of Microbiology and Infectious Diseases with Clinic, Faculty of Veterinary Medicine, University of Zagreb, 10000 Zagreb, Croatia; ljubo.barbic@vef.hr (L.B.); vladimir.stevanovic@vef.hr (V.S.); 5 Department of Epidemiology, Croatian Institute of Public Health, 10000 Zagreb, Croatia; maja.ilic@hzjz.hr; 6 Laboratory for Virology and Serology, Poultry Center, Croatian Veterinary Institute, 10000 Zagreb, Croatia; v_savic@veinst.hr; 7 Department of Anesthesiology, Reanimatology and Intensive Care Medicine, Merkur University Hospital, 10000 Zagreb, Croatia; thomas.ferenc95@gmail.com; 8 Clinic for Infectious Diseases, Clinical Hospital Center Osijek, 31000 Osijek, Croatia; dariocroatia@gmail.com (D.S.); peric@kbo.hr (L.P.); 9 Medical Faculty, Josip Juraj Strossmayer University of Osijek, 31000 Osijek, Croatia; 10 Clinic for Infectious Diseases, County Hospital Cakovec, 40000 Cakovec, Croatia; tanja.potocnik.h@gmail.com; 11 Clinic for Infectious Diseases, General Hospital Varazdin, 42000 Varazdin, Croatia; ebidvorski@gmail.com (E.D.); tbutigan@gmail.com (T.B.); 12 Environmental Health Department, Croatian Institute of Public Health, 10000 Zagreb, Croatia; kcapak@hzjz.hr; 13 Laboratory for Diagnostics, Croatian Veterinary Institute, Veterinary Institute Split, 21000 Split, Croatia; e.listes.vzs@veinst.hr; 14 Department of Virology, OIE Reference Center for West Nile Disease, Istituto Zooprofilattico Sperimentale “G. Caporale”, 64100 Teramo, Italy; g.savini@izs.it

**Keywords:** West Nile virus, immune response, neuro-invasive disease, West Nile fever

## Abstract

Data on the immune response to West Nile virus (WNV) are limited. We analyzed the antiviral cytokine response in serum and cerebrospinal fluid (CSF) samples of patients with WNV fever and WNV neuroinvasive disease using a multiplex bead-based assay for the simultaneous quantification of 13 human cytokines. The panel included cytokines associated with innate and early pro-inflammatory immune responses (TNF-α/IL-6), Th1 (IL-2/IFN-γ), Th2 (IL-4/IL-5/IL-9/IL-13), Th17 immune response (IL-17A/IL-17F/IL-21/IL-22) and the key anti-inflammatory cytokine IL-10. Elevated levels of IFN-γ were detected in 71.7% of CSF and 22.7% of serum samples (*p* = 0.003). Expression of IL-2/IL-4/TNF-α and Th1 17 cytokines (IL-17A/IL-17F/IL-21) was detected in the serum but not in the CSF (except one positive CSF sample for IL-17F/IL-4). While IL-6 levels were markedly higher in the CSF compared to serum (CSF median 2036.71, IQR 213.82–6190.50; serum median 24.48, IQR 11.93–49.81; *p* < 0.001), no difference in the IL-13/IL-9/IL-10/IFN-γ/IL-22 levels in serum/CSF was found. In conclusion, increased concentrations of the key cytokines associated with innate and early acute phase responses (IL-6) and Th1 type immune responses (IFN-γ) were found in the CNS of patients with WNV infection. In contrast, expression of the key T-cell growth factor IL-2, Th17 cytokines, a Th2 cytokine IL-4 and the proinflammatory cytokine TNF-α appear to be concentrated mainly in the periphery.

## 1. Introduction

West Nile virus (WNV) is an emerging widely distributed flavivirus. In nature, WNV is maintained in a cycle between mosquitoes (mainly of the *Culex* spp.) and animal hosts (birds), while humans represent incidental or ‘dead-end’ hosts [1]. Human WNV infections are mainly subclinical (~80%) or presented as a non-specific febrile disease (WNV fever, ~20%), however neuro-invasive disease may also occur (<1%) [2]. Central nervous system (CNS) manifestations of WNV infection include meningitis, encephalitis and poliomyelitis-like syndrome, however some atypical presentations such as cerebellitis, spastic paralysis and cranial nerve palsy are also described [3,4,5]. These manifestations are generally more prevalent in older and immunosuppressed persons [2]. While clinical manifestations and outcomes of WNV infection are well described [6], data on the immunopathogenesis of WNV infections, particularly in the context of complex cytokine immune responses, are limited.

Acute WNV infection in humans induces the synthesis of various cytokines, chemokines and growth factors that play an important role in antiviral immunity but also contribute to disease pathogenesis [7]. In the natural infection via infected mosquitoes, initial cellular targets for WNV infection in the skin include keratinocytes, Langerhans cell and dermal dendritic cells (DCs). Within the first 24 h, WNV-infected cells migrate to the skin-draining lymph nodes where the virus replicates mostly within resident DCs. Dissemination of viral progeny to permissive peripheral tissues allows virus replication in the kidney, spleen and other visceral organs as well as in immune system cells including neutrophils and monocytes. The innate immune response to WNV following natural transmission is initiated by Toll-like receptor 7 (TLR7) that, following recognition of single-stranded viral RNA, initiates the synthesis of type I interferons (IFN) as well as a variety of pro-inflammatory cytokines including TNF-α, IL-6, IL-1β and IL-12 that mediate early antiviral response. Mechanisms of specific immunity, particularly WNV-specific cytotoxic CD8+ T-cells, restrict viral replication and dissemination in the peripheral organs but, in a subset of patients, the virus enters the CNS leading to a diverse spectrum of neurological symptoms [7]. Multiple molecular pathways associated with the WNV invasion of the CNS have been proposed including: infection of the brain microvascular endothelial cells; crossing of the compromised blood brain barrier (BBB); infection of immune effector cells that migrate into the CNS upon increased expression of surface adhesion molecules (e.g., Trojan horse mechanism); infection of olfactory neurons and subsequent dissemination to the olfactory bulb; and direct axonal retrograde transport from infected peripheral neurons [8]. The ability of WNV to gain access to the CNS by crossing the BBB is mediated by the balance between proinflammatory cytokines TNF-α, IL-6, and IL-1β as well as matrix metalloproteinase 9, which disrupt the barrier, and type I and III IFN. as well as semaforin 7A that promotes BBB integrity [7]. The majority of the experimental data on the molecular mechanisms of WNV-induced neuropathogenesis are based on in vitro data and in vivo animal models showing that neurons, microglia and astrocytes represent the main cellular targets for infection in the CNS [9]. The hallmark of WNV neuroinvasive disease (WNND) pathogenesis is massive neuronal death attributed to caspase-3 and 9- mediated apoptosis involving both intrinsic and extrinsic pathways that is a result of both direct viral infection of neurons and indirect mechanisms mediated by cytotoxic factors such as proinflammatory cytokines [10,11]. The synthesis of neurotoxic factors and proinflammatory cytokines is mainly attributed to the WNV infection of microglia and recognition of a double stranded viral RNA intermediate in a TLR3-dependent manner [11]. Infection of neurons also results in the synthesis of chemokines, including CCL9, CCL10 and CCL12, that enable the recruitment of WNV-specific T-cells expressing their corresponding receptors into the CNS [7]. Despite a well-recognized role of cytokines in the immunopathogenesis of WNV infection, literature data on cytokine expression patterns in human WNV infection are limited to the analysis of pre-symptomatic and asymptomatic blood donors as well as studies focusing on persistent post-infectious syndrome associated with past WNV infection. Tobler et al. [12] showed increased levels of cytokines (IFN-α, IFN-γ, TNF-α, IL-4 and IL-10) and chemokines (CCL2, CXCL9 and CXC10) in the plasma of WNV-infected blood donors. More recently, Fares-Gusmao et al. analyzed the expression of cytokines, chemokines, soluble cytokine receptors, adhesion and apoptosis-related molecules in the plasma of 52 blood donors who were asymptomatic and eligible for donation but have subsequently tested positive for WNV RNA indicating they were at the acute viremic stage of infection. Increased levels of several cytokines including IFN-γ, IL-1β, IL-2, IL-12, IL-17, IL-10, IL-4, and IL-5 as well as chemokines CXCL8 and CXCL9 were found in the plasma of WNV-infected blood donors in comparison with WNV-negative donors [13]. Hoffman et al. reported a correlation between strong early type I interferon-mediated responses (prior to seroconversion) and a greater number of symptoms experienced by WNV-infected blood donors [14]. In addition, they reported gender-based differences in the frequency of symptoms and cytokine responses to WNV with males showing fewer symptoms and protracted synthesis of CCL2, CCL11, CXCL10 and IL-15 compared with females [15]. These results demonstrated a systemic cytokine response that is, in part, focused on the establishment of T-cell-mediated control of WNV replication even in a pre-symptomatic stage of infection. Garcia et al. [16] showed significantly higher concentrations of IFN-γ, IL-2, IL-6, IL-12p70, CXCL10 and GM-CSF in patients with a clinical diagnosis of prolonged post-infection fatigue (>6 months) reporting a history of symptomatic WNV infection. More recently, Leis et al. [17] reported elevated levels of TNF-α up to 36 months post-onset of illness in four patients with serologically-confirmed WNND with persistent post-infectious symptoms suggesting a possible role of this cytokine in the extended post-inflammatory state and long-time morbidity associated with WNV infection. Evaluation of cytokines as possible biomarkers of disease severity in WNV infection is limited to the study by Qian et al. [18] showing significantly lower serum concentrations of IL-4 in healthy persons with a history of severe versus asymptomatic infection. To the best of our knowledge, studies on cytokine expression in the serum/plasma and CSF of patients with WNV fever or WNND, particularly in the clinical context, are currently not available. 

In Croatia, human WNV infections were continuously reported in continental counties from 2012 to 2018 with several outbreaks (2012, 2013, 2017 and 2018, respectively) [19,20,21]. Phylogenetic analysis of two strains sequenced in 2013, three strains in 2017 and 8 strains in 2018 has shown circulation of WNV lineage 2 [21,22,23]. In addition, during the largest Croatian outbreak in 2018, WNV lineage 2 was detected for the first time in two goshawks (*Accipiter gentilis*) [21,23]. The majority of Croatian patients presented with WNV neuroinvasive disease, including some rare complications (WNV retinitis, myocarditis, cauda equine arachnoiditis, and opsoclonus-myoclonus syndrome) [24,25,26,27]. 

The aim of this study was to analyze the expression of 13 cytokines in the CSF and serum of patients with WNND and WNV fever in the context of clinically relevant parameters. 

## 2. Patients and Methods

### 2.1. Patients and Sample Collection

A total of 66 patients with WNV infection detected during two consecutive transmission seasons (2017–2018) were included in the study. In patients with WNND (*n* = 60), CSF, urine and serum samples were collected. Median duration of symptoms before sampling was 5 (IQR 3–9) days. In patients with WNV fever (*n* = 6), serum samples were collected. Median sampling time was 13 (IQR 11–17) days after disease onset. WNV diagnosis was confirmed according to the EU case definition for diagnosing and reporting WNV infection by detection of a) WNV RNA in CSF; b) WNV IgM antibodies in CSF or c) WNV IgM in serum confirmed by a virus neutralization test (VNT) [28].

### 2.2. WNV Detection

WNV RNA was detected in the CSF and urine samples according to the protocol of Tang et al. [29]. Briefly, viral RNA was extracted using a High Pure Viral Nucleic Acid Kit (Roche Applied Science, Mannheim, Germany). TaqMan real-time RT-PCR was performed using Brilliant III Ultra-Fast QPCR Master Mix (Agilent Technologies, Santa Clara, CA, USA) and Rotor-Gene Q real-time PCR cycler (Hilden, Germany). The thermal cycling conditions consisted of 10 min at 50 °C for reverse transcription, 3 min at 95 °C for denaturation, and 50 cycles of 15 s at 95 °C and 1 min at 60 °C for amplification. Samples identified as positive using the real-time RT-PCR assay were subjected to conventional RT-PCR using pan-flavi primers targeting the NS5 gene (FP: 5′-TACAACATGATGGGVAARAGAGAGA-3′, RP: 5′-AGCATGTCTTCYGTBGTCATCCAYT-3′) to amplify 1,085 bp product [30] by use of PrimeScript^TM^ One Step RT-PCR Kit Ver.2 (Takara Bio Inc, Kusatsu, Japan). The thermal cycling conditions consisted of 30 min at 50 °C for reverse transcription, 2 min at 94 °C for denaturation, and 40 cycles of 30 s at 94 °C, 30 s at 60 °C and 1 min at 72 °C, completed by a final extension of 10 min at 72 °C. For samples that yielded faint PCR product, nested PCR using WNV internal primers (FP: 5′-AGAGAGAAGAAGCCTGGAGAG-3′, RP: 5′-CTTTGGTGATGCGTGTGTC-3′) amplifying 262 bp product was performed [21] by use of EmeraldAmp MAX PCR Master Mix (Takara Bio Inc). The thermal cycling conditions were the same as for the RT-PCR except for the exclusion of the reverse transcription 30 min at 50 °C. All conventional RT-PCR and PCR reactions were conducted on Biometra T3000 PCR Cycler (Biometra, GmbH, Göttingen, Germany) with a final primer concentration of 1 µM. Amplified products were Sanger sequenced in both directions by Humanizing Genomics, Macrogen Inc. (Amsterdam, The Netherlands) with the use of the internal primers. The obtained nucleotide sequences were confirmed as WNV specific using BLASTn algorithm at the National Center for Biotechnology Information (NCBI) website (http://www.ncbi.nlm.nih.gov, accessed on 10 October 2018).

WNV IgM antibodies in serum and CSF samples were detected using a commercial enzyme-linked immunosorbent assay (ELISA; Euroimmun, Lübeck, Germany) and interpreted as follows: ratio < 0.8 negative, 0.8–1.1 borderline, ≥1.1 positive. VNT was performed at the OIE Reference Center for West Nile Disease, Istituto Zooprofilattico Sperimentale “G. Caporale”, Teramo, Italy. The WNV antibody titre was defined as the reciprocal value of the highest serum dilution that showed 100% neutralization. Titer of ≥10 was considered positive. Prior to VNT, WNV antigen (strain Eg-101) was titrated by 50% TCID (TCID_50_) using Vero cells. After four days, the titre was determined using the Reed and Muench formula [31].

### 2.3. Antiviral Cytokine Response

Antiviral cytokine response in the serum and CSF was analyzed by a multiplex bead based assay for the simultaneous quantification of 13 human antiviral cytokines (LEGENDplex Human Th cytokine panel, BioLegend, San Diego, CA, USA) on a FACS Canto II flow cytometer (Beckton Dickinson, USA) [32,33]. Serum and CSF samples were stored in aliquots to avoid freeze-and-thaw cycles at −80 °C until analysis. In order to provide a comprehensive analysis of cytokine responses (particularly those related to T-cell immunity), we analyzed a cytokine panel associated with innate and early pro-inflammatory immune responses (TNF-α, IL-6), Th1 type immune response (IL-2, IFN-γ), Th2 immune response (IL-4, IL-5, IL-9 and IL-13), Th17 immune response (IL-17A, IL-17F, IL-21 and IL-22) and the key anti-inflammatory cytokine IL-10. Cytokine concentrations (pg/mL) in healthy controls (*n* = 16) measured by LEGENDPlex panel were: IL-5 (mean 3.9, range non-detectable, ND-12.7, detectable in 69% of samples); IL-13 (mean 5.1, range 1.4-17.3, detectable in all samples); IL-2 (mean 39.1, range ND-79.4, detectable in 25% of samples); IL-6 (mean 12.9, range ND-18.4, detectable in 56% of samples); IL-9 (mean 3.9, range ND-18.4, detectable in 25% of samples); IL-10 (mean 1.1, range ND-1.3, detectable in 19% of samples); IFN-γ (mean 11.5, range ND-39.4, detectable in 63% of samples); TNF-α (mean 5.9, range ND-14.0, detectable in 50% of samples); IL-17A (not detectable), IL-17F (mean 29.0, range ND-107.0, detectable in 50% of samples); IL-4 (mean 18.1, ND-56.5, 44% of samples); IL-22 (mean 6.4, range ND-15.2, detectable in 38% of samples), IL-21 not available [34].

### 2.4. Statistical Analysis

Study participants are described by age, gender and clinical presentation. Numerical variables are expressed as medians and interquartile ranges (IQR). Categorical variables are expressed as frequencies and percentages, with 95% confidence intervals (CI). Mann-Whitney U test was used to compare age between males and females, and Kruskal-Wallis test to compare age of patients by clinical presentation. To identify possible correlation with cytokine levels, several parameters were taken into consideration: age, gender, clinical presentation (fever, meningitis, meningoencephalitis), CSF/blood laboratory parameters and sampling time. The proportion of serum and CSF samples with positive cytokine response was calculated. The Fisher exact test was used to compare a difference in the proportion of positive cytokine between serum and CSF samples. The cytokine levels in paired CSF and serum were compared using Wilcoxon matched-pairs signed rank test. The association between cytokine levels and age, days after disease onset, clinical diagnosis, blood leukocyte count, CSF leukocyte count, protein levels and percentage of lymphocytes/neutrophils was assessed with Spearman’s rho rank-based correlation with Bonferroni adjustment for multiple comparisons. Adjusted *p* values <0.05 were considered significant. Statistical analysis was performed using Stata, version 16 software.

## 3. Results

Basic demographic and clinical characteristics of study participants are presented in Table 1 and Table 2. In the tested group, there were 39 (59.1%) males (median age 66 years, IQR 61–76) and 27 (40.9%) females (median age 61 years, IQR 53–76). Clinical presentations were febrile disease (WNV fever, *n* = 6), meningitis (*n* = 36) and meningoencephalitis (*n* = 24). Patients with neuroinvasive disease were older (meningitis median age 64.5 years, IQR 56.5–76; meningoencephalitis median age 71 years, IQR 63.5–76) compared to patients with WNV fever (median age 50 years, IQR 40–66, *p* = 0.01).

For 37 (56.1%) patients with WNND, matched serum and CSF samples were analyzed while for 29 (43.9%) patients (23 WNND, 6 WNV fever) only serum samples were available. Laboratory analysis of the CSF samples is presented in Table 3. Significantly higher CSF protein levels were found in patients with meningoencephalitis (median 1.31 g/L, IQR 0.9–1.56) compared to patients with meningitis (median 0.75 g/L; IQR 0.615–1.025, Mann Whitney U test *p* = 0.007).

**Figure 1:**
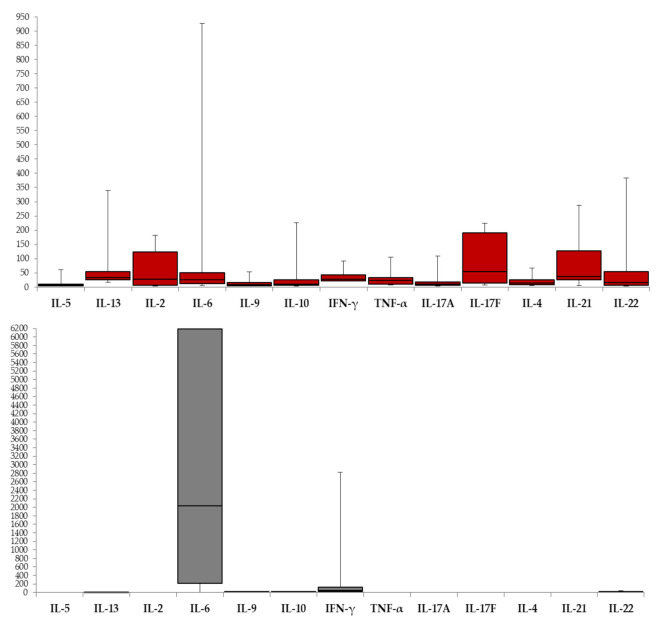
Cytokine expression pattern (pg/mL) in serum (red box plots) and CSF (grey box plots) in patients with WNV infection. Boxes represent median and interquartile range. Error bars indicate the minimum and maximum values.

Proportion of samples with elevated cytokine level is presented in Table 4. Comparing the serum and CSF results, elevated levels of IFN-γ were detected in 71.7% of CSF samples and 22.7% of serum samples (Fischer exact test *p* = 0.003). Expression of IL-2, IL-4, TNF-α and Th1 17 cytokines (IL-17A, IL17F, IL-21) was detected in the serum but not in the CSF (except one positive CSF sample for IL-17F and IL-4) (Figure 1). While IL-6 levels were markedly higher in the CSF samples (median 2036.71, IQR 213.82–6190.5) compared to serum samples (median 24.48, IQR 11.93–49.81; *p* < 0.001), no significant difference in the IL-13, IL-9, IL-10, IFN-γ and IL-22 levels in serum and CSF samples was found. Serum cytokine concentrations (including IL-6 and IFN-γ) in healthy controls [34] were lower compared with WNV patients (Table 4) except for IL-4 (range in healthy controls ND-56.5 pg/mL) vs. WNF patients (8.17–28.11 pg/mL).

Correlation of serum and CSF cytokine levels and patient’s age, date of sampling and clinical presentation is presented in Table 5. A significant positive correlation between serum IL-6 levels and age was found (rho = 0.407, *p* = 0.036). Levels of other tested cytokines showed no correlation with age and date of sampling. In addition, serum cytokine levels did not differ among patients with WNV fever and patients with meningitis/meningoencephalitis.

In order to evaluate the possible association between local cytokine immune responses and both local as well as systemic inflammatory responses, we compared CSF cytokine levels with selected laboratory parameters in the CSF and periphery. A significant positive correlation between IFN-γ and CSL leukocyte count (rho = 0.706, *p* = 0.011) as well as blood leukocyte count (rho = 0.664, *p* = 0.028) was observed as shown in Table 6.

## 4. Discussion

The results of this study have shown, for the first time, cytokine responses to WNV infection in a cohort of patients with WNV fever and WNND presenting with meningitis or meningoencephalitis. The WNND cytokine pattern is characterized by exceptionally high intrathecal synthesis of IL-6, lack of significant differences in the serum vs. CSF concentrations of IL-13, IL-9, IL-10, IFN-γ and IL-22 as well as by the absence of IL-2, IL-4, TNF-α and Th17 cytokines (IL-17A, IL-17F and IL-21) in the CSF of WNV-infected persons.

Innate immune responses in neuro-invasive WNV infections are initiated by the recognition of a double stranded WNV RNA replication intermediate by TLR-3 on microglia that results in the synthesis of IL-6 and other proinflammatory cytokines [8]. IL-6 is a pleiotropic cytokine that plays a neuroprotective role by promoting oligodendrocyte differentiation, mediating regeneration of peripheral nerves and acting as a neurotrophic factor [29]. However, IL-6 trans-signaling pathway (mediated by an interaction between IL-6/soluble IL-6R complex with gp130 subunit) has been implicated in the pathogenesis of malignant, inflammatory and autoimmune diseases [35]. IL-6 has been evaluated as a CSF neuroinflammatory biomarker in a diverse spectrum of human diseases but has not been previously described in WNND [35,36,37,38]. Therefore, the precise contribution of IL-6 in the context of neuroprotection vs. neuroinflammation remains to be determined. 

Our study also reported high CSF concentrations of IFN-γ, the principal cytokine involved in the regulation of both innate and adaptive immunity. In vivo studies on mouse WNV models provided direct evidence on the important role of IFN-γ in immunological mechanisms that are responsible for the control of viral replication in the CNS, e.g., antiviral activity of γδ cells, WNV-specific cytotoxic CD8+ T-cells and Th1 type cytokine responses in CD4+ T-cells [39,40]. Therefore, high intrathecal concentration of IFN-γ in the CSF of WNND probably plays an important part in immune-mediated suppression of viral replication. 

The role of Th17-type cytokines in the pathogenesis of neuroinvasive flavivirus infections has mostly been considered in the context of neutrophil infiltrations. Experiments in WNV mice models by Acharya et al. [41] showed that IL-17A protects mice from lethal WNV infection by promoting expression of genes associated with CD8+ T-cell cytotoxicity. In addition, treatment with recombinant IL-17A reduced the kinetics of viral replication and increased survival in infected mice suggesting a potential protective role for this cytokine in WNV infection. Very low serum levels of IL-17A as well as complete absence of IL-17A expression in the CSF possibly suggest dysregulation of IL-17A expression in human WNND. Studies in knock-out mice showed that a Th17 cytokine IL-22 exacerbates lethal WNV encephalitis by promoting WNV neuro-invasion by modulation of the chemokine network (in particular CXCR2) expression that regulates neutrophil migration into the CNS [42]. According to our results, IL-22 was the only Th17 cytokine detectable in the CSF suggesting its possible contribution to the immunopathology of WNND. Since this is the first study providing a comprehensive analysis of cytokine expression in the serum of patients with WNF as well as in paired serum/CSF samples of patients with WNND, our results can only be indirectly compared with data on serum/plasma cytokine expression in pre-symptomatic and asymptomatic WNV-infected person blood donors. 

Tobler et al. [12] analyzed cytokine and chemokine expression in the plasma of blood donors that tested positive for WNV RNA prior to and after anti-WNV IgM seroconversion and compared it with WNV-negative donors. Increased concentrations of IFN-α, IFN-γ, IL-4, IL-10, TNF-α, CCL2, CXCL9 and CXCL10 have been found in acute-phase viremic samples obtained prior to IgM seroconversion. Following IgM seroconversion, concentrations of IFN-γ, IL-4, IL-10, TNF-α, CCL2, CXCL9, and CXCL10 were increased compared with controls. Our results have also shown the presence of a similar set of cytokines including IFN-γ, IL-4, IL-10, and TNF-α in the serum of patients with WNV fever and WNND. Fares-Gusmao et al. [13] investigated cytokines as a part of immune marker profiles in acute, viremic pre-symptomatic/asymptomatic blood donors subsequently diagnosed with flavivirus infections including dengue virus (DENV), WNV and Zika virus (ZIKV) as well as in controls. A comprehensive analysis of biological response modifiers in the plasma of WNV-infected blood donors showed increased concentrations of cytokines (IFN-γ, IL-1β, IL-2, IL-12, IL-17, IL-10, IL-4, and IL-5), soluble cytokine receptors (IL-2R and ST2/IL-1R4), granulocyte macrophage colony stimulating factor GM-CSF, and chemokines (CXCL8 and CXCL9), as well as decreased levels of several apoptosis-related molecules. The results of these two studies suggest that cytokines associated with Th1 (IL-2), Th2 (IL-4, IL-5), Th17 and anti-inflammatory responses (IL-10) that were analyzed in our study on symptomatic WNV-infected patients also represent an important part of early immune response to WNV in viremic pre-symptomatic/asymptomatic patients.

In addition, Fares-Gusmao et al. [13] identified a common immune signature for asymptomatic infections with DENV, WNV and ZIKV that included increased levels of IL-12, IL-17, IL-5, IL-10, CXCL9, E-Selectin and ST2/IL-1R4 as well as decreased levels of IL-13 and CD40 when compared to controls. These results suggest that cytokines could be further evaluated as possible surrogate biomarkers for differentiation of early flavivirus infections before seroconversion.

Qian et al. [18] evaluated a panel of immune markers associated with host’s susceptibility to severe clinical course of WNV infection in a cohort of healthy adults reporting a history of asymptomatic or severe WNV infection. Analysis of individual variations in levels of antibodies, serum concentration of cytokines and genome-wide transcriptional profiles of peripheral blood mononuclear cells showed significantly decreased concentrations of serum IL-4 as well as altered gene expression patterns. The results of this study suggest a possible contribution of IL-4, one of the key Th2 cytokines, in natural resistance to WNV infection. Our study reported detectable IL-4 in the serum of patients with both WNV fever and WNND. However, IL-4 was not detected in the CSF of WNND patients suggesting it does not play an important role in local immune response to WNV.

The majority of literature data on the expression of cytokines and other inflammatory mediators in the CSF and serum of patients with neuroinvasive arboviral infections are focused on tick-borne encephalitis (TBE) and Toscana virus (TOSV) neuroinvasive disease. 

In our recent report on clinical, virological and immunological findings in three patients with neuroinvasive TOSV infection, significantly increased concentrations of IL-6, IFN-γ and IL-10 in the CSF compared to serum have been described [43]. Our results in WNND showed a reverse pattern of serum/CSF expression, possibly suggesting that lower expression of IL-10 as an anti-inflammatory cytokine impairs the homeostatic immune mechanisms related to the control of local immune response in the WNV CNS infection. Cytokine expression patterns in the CSF and serum of TBE patients described by Günther et al. [44] showed important similarities with our results, particularly in the context of increased levels IFN-γ and IL-6 as well as decreased/undetectable concentrations of TNF-α in the CSF. Recently, Bogovič et al. [45] showed significantly higher expression of mediators associated with innate and Th1 type of immune responses in the CSF, including IL-8, IFN-γ, CXCL9 and CXCL10, that positively correlated with disease severity. In contrast, concentrations of Th17 cytokines and mediators associated with humoral immunity were higher in the serum compared with the CSF and failed to correlate with clinical presentation of disease. In contrast to our data on WNND, Grygorczuk et al. [46] reported high intrathecal expression of Th17 cytokines in TBE suggesting a difference in the cytokine CSF profiles in the two neuroinvasive arboviral infections.

Cytokine measurement in the CSF at a single time point versus monitoring trends of cytokine expression over time, represents an important but well-established limitation of neuro-immunological studies conducted in humans. Therefore, questions regarding the timelines and kinetics of CSF cytokine responses observed in our study can only be further addressed in animal models.

Some limitations of this study should be taken into account when interpreting the results. Due to limited availability of CSF specimens from healthy individuals, also related to ethical implications, there was no control group to compare the results. Therefore, our findings could not be controlled for different confounders. However, cytokine responses in WNV patients were compared with literature data on cytokine expression in healthy persons determined by the identical methodological approach. 

A variety of factors can influence the patterns of cytokine expression in humans, including age, gender-related hormonal and genetic differences in the immune system, other epigenetic and genetic factors (including cytokine gene polymorphism), co-morbidities and microbiome [47,48]. Advanced age is a well-established risk factor in WNV infection associated with susceptibility to severe disease and this association is, at least in part, associated with dysregulation of cytokine responses and alteration of immune cell functions during immuno-senescence [49]. Kong et al. showed downregulated TLR3 expression on macrophages in WNV-infected young persons. However, TLR3 expression remained elevated in elderly persons leading to elevated production of proinflammatory cytokines and sustained high expression of IL-6 and IFN-β1 [50]. These data suggest that investigations of age-related differences in both cytokine and cellular immune responses in WNV infection, particularly in the clinical context, warrant further investigations. 

## 5. Conclusions

The results of this study have shown a well-defined pattern of cytokine expression in human WNV fever and WNND. The possible role of IL-6 as a biomarker in WNND remains to be determined.

## Figures and Tables

**Table 1:** Demographic characteristic of patients with WNV disease.

Characteristic	N Tested (%)	95%CI	Median, Years (IQR)
Male	27 (40.9)	29.57–53.30	61 (53–76)
Female	39 (59.1)	46.69–70.43	66 (61–76)
Total	66 (100)		

Mann-Whitney U test *p* = 0.13; IQR = interquartile range.

**Table 2:** Clinical diagnosis of patients with WNV disease.

Clinical Diagnosis	N Tested (%)	95%CI	Median, Years (IQR)
WNV fever	6 (9.1)	4.08–19.04	52 (40–66)
Meningitis	36 (54.5)	42.28–66.28	64.5 (56.5–76)
Meningoencephalitis	24 (36.4)	25.53–48.79	71 (63.5–76)
Total	66 (100)		

Kruskal-Wallis test *p* = 0.01; IQR = interquartile range.

**Table 3:** Laboratory findings in patients with WNV neuroinvasive disease.

Parameter	Median	IQR	Reference Values
Blood leukocyte count (×10^12^)	9.4	7.2–11.2	3.4–9.7
Cerebrospinal fluid (CSF) leukocyte count	484	95–736	<5
CSF proteins (g/L)	0.945	0.691–1.300	0.17–0.37
CSF Neutrophils (%)	47	20–58	
CSF Lymphocytes (%)	53	42–76	

IQR = interquartile range.

**Table 4:** Cytokine levels in serum and CSF samples of patients with WNV infection.

Cytokine	WNV Serum (*n* = 66)			WNV CSF (*n* = 37)			*p*
	N Positive (%)	Median (pg/mL)	IQR	N Positive (%)	Median (pg/mL)	IQR	
IL-5	32 (48.5)	6.70	4.91–10.42	1 (2.6)	NA	NA	NA
IL-13	11 (16.7)	33.26	24.65–54.6	3 (7.9)	9.34	2.41–9.34	NA
IL-2	8 (12.1)	27.48	5.70–123.71	0	NA	NA	NA
IL-6	61 (92.4)	24.48	11.93–49.81	35 (92.1)	2036.71	213.82–6190.5	<0.001
IL-9	24 (36.4)	7.70	4.38–15.51	25 (65.8)	5.34	3.57–8.35	0.25
IL-10	18 (27.3)	10.74	5.75–25.18	17 (44.7)	5.94	4.06–10.54	0.62
IFN-γ	15 (22.7)	27.22	20.95–41.82	27 (71.1)	49.59	23.07–127.37	1.00
TNF-α	17 (25.8)	22.69	9.99–32.35	0	NA	NA	NA
IL-17A	38 (57.6)	9.44	6.11–17.42	0	NA	NA	NA
IL-17F	12 (18.2)	53.84	13.68–190.17	1 (2.6)	NA	NA	NA
IL-4	16 (24.2)	13.68	8.17–26.11	1 (2.6)	NA	NA	NA
IL-21	17 (25.8)	36.56	24.89–127.32	0	NA	NA	NA
IL-22	18 (27.3)	15.75	6.37–53.44	20 (52.6)	5.58	3.62–11.86	0.07

Wilcoxon matched-pairs signed-rank test; IQR = interquartile range; NA = not applicable.

**Table 5:** Comparison of serum and CSF cytokine levels and patient’s age, sampling time and clinical diagnosis.

Sample	Cytokine (pg/mL)	Age (Years)			Days after Disease Onset			Clinical Diagnosis		
		rho	*p*	*p* adj.	rho	*p*	*p* adj.	rho	*p*	*p* adj.
Serum	IL-5	0.195	0.411	1	−0.454	0.044	0.266	0.511	0.021	0.128
	IL-13	0.464	0.354	1	−0.058	0.913	1	0.488	0.326	1
	IL-2	−0.342	0.452	1	−0.273	0.554	1	0.144	0.757	1
	IL-6	0.407	0.006	0.036	0.111	0.471	1	0.279	0.066	0.396
	IL-9	0.000	1	1	−0.387	0.195	1	0.290	0.336	1
	IL-10	0.063	0.845	1	0.239	0.456	1	0.190	0.553	1
	IFN-γ	0.207	0.593	1	−0.219	0.571	1	0.367	0.332	1
	TNF-α	−0.551	0.063	0.379	−0.228	0.476	1	0.235	0.461	1
	IL-17A	0.079	0.692	1	0.116	0.563	1	0.067	0.739	1
	IL-17F	0.308	0.420	1	−0.366	0.333	1	0.733	0.024	0.147
	IL-4	0.384	0.218	1	−0.357	0.255	1	0.291	0.358	1
	IL-21	0.398	0.225	1	−0.213	0.528	1	0.290	0.386	1
	IL-22	0.182	0.552	1	−0.360	0.309	1	0.475	0.101	0.606
CSF	IL-6	0.281	0.125	0.752	0.235	0.203	1	0.054	0.772	1
	IL-9	0.209	0.348	1	0.141	0.532	1	−0.194	0.386	1
	IL-10	0.044	0.877	1	0.221	0.429	1	0.159	0.569	1
	IFN-γ	0.016	0.940	1	−0.054	0.800	1	0.197	0.356	1
	IL-22	0.153	0.544	1	−0.209	0.403	1	−0.187	0.458	1

Spearman’s correlation coefficient adjusted for multiple comparisons (Bonferroni); CSF IL-5, IL-13, IL-2, TNF-α, IL-17A, IL-17F, IL-4, IL-21 not applicable.

**Table 6:** Comparison of CSF cytokine levels and CSF/blood laboratory findings.

Cytokine	Blood Leukocyte Count			CSF Leukocyte Count			CSF Protein Level			CSF % Neutrophils			CSF % Lymphocytes		
	rho	*p*	*p* adj.	rho	*p*	*p* adj.	rho	*p*	*p* adj.	rho	*p*	*p* adj.	rho	*p*	*p* adj.
IL-6	0.143	0.503	1	0.552	0.005	0.077	0.322	0.125	1	0.423	0.039	0.585	−0.437	0.033	0.490
IL-9	0.263	0.307	1	0.178	0.495	1	0.185	0.477	1	0.321	0.209	1	−0.327	0.199	1
IL-10	0.218	0.519	1	0.654	0.029	0.433	0.245	0.467	1	0.409	0.211	1	−0.427	0.189	1
IFN-γ	0.664	0.001	0.028	0.706	0.001	0.011	0.101	0.681	1	0.041	0.868	1	−0.340	0.889	1
IL-22	−0.218	0.455	1	−0.226	0.436	1	−0.072	0.805	1	−0.271	0.349	1	0.283	0.326	1

## Data Availability

Not applicable.
